# MYBBP1A suppresses breast cancer tumorigenesis by enhancing the p53 dependent anoikis

**DOI:** 10.1186/1471-2407-13-65

**Published:** 2013-02-07

**Authors:** Kensuke Akaogi, Wakana Ono, Yuki Hayashi, Hiroyuki Kishimoto, Junn Yanagisawa

**Affiliations:** 1 Graduate School of Life and Environmental Sciences, University of Tsukuba, Tsukuba Science City, Ibaraki, 305-8577, Japan; 2 Life Science Center of Tsukuba Advanced Research Alliance, University of Tsukuba, Tsukuba Science City, Ibaraki, 305-8577, Japan

**Keywords:** Breast cancer, Tumorigenesis, Anoikis, p53, MYBBP1A

## Abstract

**Background:**

Tumor suppressor p53 is mutated in a wide variety of human cancers and plays a critical role in anoikis, which is essential for preventing tumorigenesis. Recently, we found that a nucleolar protein, Myb-binding protein 1a (MYBBP1A), was involved in p53 activation. However, the function of MYBBP1A in cancer prevention has not been elucidated.

**Methods:**

Relationships between *MYBBP1A* expression levels and breast cancer progression were examined using patient microarray databases and tissue microarrays. Colony formation, xenograft, and anoikis assays were conducted using cells in which *MYBBP1A* was either knocked down or overexpressed. p53 activation and interactions between p53 and MYBBP1A were assessed by immunoprecipitation and western blot.

**Results:**

*MYBBP1A* expression was negatively correlated with breast cancer tumorigenesis. *In vivo* and *in vitro* experiments using the breast cancer cell lines MCF-7 and ZR-75-1, which expresses wild type p53, showed that tumorigenesis, colony formation, and anoikis resistance were significantly enhanced by *MYBBP1A* knockdown. We also found that MYBBP1A binds to p53 and enhances p53 target gene transcription under anoikis conditions.

**Conclusions:**

These results suggest that MYBBP1A is required for p53 activation during anoikis; therefore, it is involved in suppressing colony formation and the tumorigenesis of breast cancer cells. Collectively, our results suggest that MYBBP1A plays a role in tumor prevention in the context of p53 activation.

## Background

Breast cancer is the most commonly occurring cancer in women worldwide. It has been estimated that more than 1.6 million new cases of breast cancer occurred in 2010 [1]. Cancer cells develop features that are fundamentally different from those of normal cells. One hallmark of cancer cells is their ability to survive and proliferate in the absence of extracellular matrix (ECM)-derived signals [2].

The tumor suppressor p53 plays a central role in coordinating the responses to stresses induced by a wide array of stimuli. Under normal conditions, cellular p53 protein levels are maintained at basal levels. However, in response to genotoxic stresses, such as exposure to ultraviolet light or γ-irradiation, p53 protein levels increase and trigger either cell cycle arrest or apoptosis [3]. p53 plays a critical role in cancer prevention, because p53 can suppress tumorigenesis by inducing cell cycle arrest and apoptosis through its transcriptional activity. p53 is one of the tumor suppressor genes that is most frequently found to be inactivated in cancer [4].

p53 also plays a critical role in anoikis. Anoikis, defined as detachment-induced apoptosis [5], reflects the essential requirement of most normal epithelial cells for ECM-derived survival signals [6]. When these signals are denied, for example, upon detachment and continued culture in suspension or in soft agar, cells will rapidly undergo cell cycle arrest and apoptosis. The capacity of cancer cells for anchorage-independent growth under conditions of detachment and suspension in soft agar correlates well with their tumorigenic potential [2].

p53 is required for anoikis in many cell types [7], including epithelial cells [8-12]. Under detached conditions, p53 enhances *p21*, *Bax*, and *PUMA* transcription and induces cell cycle arrest and apoptosis [9,11]. However, the signaling pathways that regulate p53-dependent anoikis are largely unknown.

Recently, we found that a nucleolar protein, Myb-binding protein 1a (MYBBP1A), was involved in p53 activation. When cells were exposed to cellular stresses, MYBBP1A translocated from the nucleolus to the nucleoplasm. The translocated MYBBP1A promoted p53 acetylation and accumulation by facilitating the interaction between p53 and histone acetyltransferase p300; thus, MYBBP1A could enhance p53 target gene transcription [13]. Previous studies revealed that MYBBP1A was involved in regulating intracellular energy status, inflammation, and myogenesis [14-16]. In addition, Sanhueza *et al.* recently reported that MYBBP1A regulates the proliferation and migration of head and neck squamous cell carcinoma cells [17]. However, the role of MYBBP1A in breast cancer prevention and the detailed mechanisms underlying these activities have not been determined.

In this study, we show that *MYBBP1A* expression is associated with breast cancer tumorigenesis through an extensive analysis of the Oncomine database. *In vitro* and *in vivo* experiments using the breast cancer cell lines, which expresses wild type p53, revealed that tumorigenesis, colony formation, and anoikis resistance were significantly enhanced by *MYBBP1A* knockdown. MYBBP1A binds to p53 under detached conditions and enhances p53 target gene transcription, as evidenced by co-immunoprecipitation experiments and RT-qPCR. These results suggest the physiological significance of MYBBP1A in p53 activation and cancer prevention.

## Methods

### Cell culture and transfection

MCF-10A, human mammary epithelial cells, were maintained in Dulbecco’s modified Eagle’s medium-F12 (DMEM-F12) (Invitrogen, Carlsbad, CA) supplemented with 0.5 μg/ml hydrocortisone (Sigma-Aldrich, St Louis, MO), 10 μg/ml insulin (Sigma-Aldrich, St Louis, MO), and 20 ng/ml recombinant human EGF (Peprotech, Rocky Hill, NJ). MCF-7 human breast cancer cells were maintained in DMEM (Sigma-Aldrich, St Louis, MO). ZR-75-1 human breast cancer cells were maintained in RPMI 1640 (Nacalai Tesque, Kyoto, Japan). All media were supplemented with 10% fetal bovine serum (FBS) and 1% penicillin–streptomycin solution (Nacalai Tesque, Kyoto, Japan). Transfection was performed using Lipofectamine LTX (Invitrogen, Carlsbad, CA).

### Expression vectors and antibodies

cDNAs encoding full-length *p53* and *MYBBP1A* were amplified using PCR and subcloned into pcDNA3 plasmids (Invitrogen, Carlsbad, CA) and pQCXIP (Clontech, Mountain View, CA) containing sequences encoding FLAG sequences. Anti-β-Actin (Sigma-Aldrich, St Louis, MO) and anti-human-p53 (Santa Cruz, Santa Cruz, CA) monoclonal antibodies and rabbit anti-p53-K382Ac(Cell Signaling Technology, Danvers, MA) polyclonal antibody were used according to the manufacturers’ instructions. Rabbit anti-human MYBBP1A antibody was raised against a synthetic peptide corresponding to amino acids 1265–1328 of human MYBBP1A.

### Oncomine analysis

The Oncomine database and gene microarray analysis tool, a repository for published complementary DNA microarray data [18,19], were explored (July 2012) for MYBBP1A mRNA expression in non-neoplastic and breast cancer tissues. Statistical analysis of the differences in MYBBP1A expression between these tissues used Oncomine algorithms, which provided multiple comparisons among different studies [20-22]. Data sets obtained from TCGA Breast, Finak Breast, and Richardson Breast 2 included various stage, and all cancer samples were invasive.

### Immunohistochemistry (IHC)

Human breast cancer tissue microarrays were purchased from SuperBioChips Laboratories (Seoul, Korea) included various stages, and all cancer samples were invasive. Formalin-fixed tissues were dewaxed in xylene and rehydrated in alcohol. For antigen retrieval, the sections were subsequently heated in EDTA buffer (1 mM, pH 8.0) in a microwave oven for 5 min. Endogenous peroxidase activity was suppressed using a solution of 3% hydrogen peroxide in methanol for 6 min. The samples were stained with avidin–biotin–peroxidase complexes using a Histofine SAB-PO Immunohistochemical Staining Kit (Nichirei, Tokyo, Japan) according to the manufacturer’s instructions. Rabbit anti-MYBBP1A antibody was used at a dilution of 1:50.

### RNA interference

Methods for stable RNA interference followed those described by Kajiro *et al.*[23]. To generate a shRNA retroviral supernatant, GP2-293 cells (Clontech, Mountain View, CA) were co-transfected with a p10A1 vector encoding an envelope protein and pRETRO-SUPER (Oligo Engine, Seattle, WA) vector containing either the MYBBP1A or luciferase (control) target sequence. MCF-7 cells were incubated with the retroviral supernatant in the presence of 8 μg/ml polybrene. Infected cells were selected using 1 μg/ml of puromycin. The target sequences were 5^′^- TTCAGGGCATATTTCATCTCGGACC-3^′^ for MYBB1A #1, 5^′^-TTCAGGGCATATTTCATCTCGGACC-3^′^ for MYBBP1A #2, and 5^′^-GAAGCTGCGCGGTGGTGTTGT-3^′^ for luciferase. For siRNA transfection, cells at 30%–50% confluency were transfected using Lipofectamine RNAi MAX (Invitrogen, Carlsbad, CA) according to the manufacturer’s protocol. All siRNAs were purchased from Invitrogen. The siRNA duplexes were MYBBP1A, 5^′^-UCUUUCAGUCAGGUCGGCUGGUGAA-3^′^ and p53, 5^′^-CCAGUGGUAAUCUACUGGGACGGAA-3^′^. Stealth RNAi negative control was used as a negative control.

### Tumor xenograft models

All animal experiments were performed in accordance with institutional guidelines. The tumor xenograft models have been described previously [23]. Each mouse was subcutaneously injected with 100 μl of cell suspension (5 × 10^6^) in both flanks. At the time points indicated in the figures, the tumors were excised, weighed, and fixed or stored in liquid nitrogen.

### Soft agar colony-formation assay

For soft agar assays, 22,000 cells were mixed with 1 ml of 0.35% top agar and plated onto a 35-mm six-well plate containing bottom plugs (0.6% agar, 10% FBS, 1× DMEM). After the top agar had solidified (about 2 h at 37°C), 200 μl of DMEM was added into each well to prevent dehydration. This covering medium was changed every 2 or 3 days during culture. After allowing growth for 2 weeks at 37°C, colonies with a diameter of >100 μm were counted.

### Anoikis assay

MCF-10A cells were plated at a density of 3 × 10^5^ cells per well in six-well plates in an ultra-low attachment culture dish (Corning, Tewksbury, MA) and maintained in DMEM-F12 (Invitrogen, Carlsbad, CA) supplemented with 10% FBS, 0.5 μg/ml hydrocortisone (Sigma-Aldrich, St Louis, MO), 10 μg/ml insulin (Sigma-Aldrich, St Louis, MO), and 20 ng/ml recombinant human EGF (Peprotech, Rocky Hill, NJ). MCF-7 cells were plated at a density of 3 × 10^5^ cells per well in DMEM with 5% FBS. ZR-75-1 cells were plated at a density of 3 × 10^5^ cells per well in RPMI 1640 with 10% FBS. All media were supplemented with 1% penicillin–streptomycin solution (Nacalai Tesque, Kyoto, Japan). Before analysis, the samples were treated with trypsin to disperse the cells. Cell viability after detachment was determined by trypan blue dye exclusion. Viable cells were determined by MTT assay using an MTT cell counting kit (Nacalai Tesque, Kyoto, Japan). Apoptotic cell proportion was determined by fluorescence-activated cell sorting (FACS) analysis using PI and fluorescein isothiocyanate (FITC)-conjugated Annexin V (MBL, Tokyo, Japan) staining.

### Real-time RT-PCR

Real-time RT-PCR was performed as described previously [24]. Cells were homogenized in 1 ml Isogen (Nippon Gene, Tokyo, Japan), and the total RNA was extracted according to the instruction manual. cDNA was synthesized from total RNA using ReverTra Ace reverse transcriptase (Toyobo, Osaka, Japan) and oligo dT primers. Real-time PCR was used to amplify fragments representing the indicated mRNA expressions. The primer sequences used were as follows: 

GAPDH fw primer: 5^′^- GTATGACTCCACTCACGGCAAA -3^′^

GAPDH rv primer: 5^′^-GGTCTCGCTCCTGGAAGATG-3^′^

p21 fw primer: 5^′^-GGAGACTCTCAGGGTCGAAA-3^′^

p21 rv primer: 5^′^-TTAGGGCTTCCTCTTGGAGA-3^′^

Bax fw primer: 5^′^-AGCAAACTGGTGCTCAAGG -3^′^

Bax rw primer: 5^′^-CTTGGATCCAGCCCAACAG -3^′^

PUMA fw primer: 5^′^-GGGCCCAGACTGTGAATCCT-3^′^

PUMA rv primer: 5^′^-ACGTGCTCTCTCTAAACCTATGCA-3^′^

### Co-immunoprecipitation and immunoblotting

Cells were lysed in TNE buffer [10 mM Tris–HCl (pH 7.8), 1% Nonidet P-40 (NP-40), 0.15 M NaCl, 1 mM ethylenediaminetetraacetic acid (EDTA), 1 M phenylmethylsulfonyl fluoride (PMSF), 1 g/ml aprotinin]. The extracted proteins were immunoprecipitated with antibody-coated protein G Sepharose beads (GE Healthcare Japan, Tokyo, Japan). Bound proteins were separated by SDS–PAGE, transferred to polyvinylidene difluoride membranes (Millipore, Temecula, CA), and detected with appropriate primary antibodies and horseradish peroxidase-conjugated secondary antibodies. Specific proteins were visualized using an enhanced chemiluminescence (ECL) western blot detection system (GE Healthcare Japan, Tokyo, Japan).

### Chromatin immunoprecipitation (ChIP) and real-time PCR detection

ChIP assay was performed according to the published procedures [24]. The primers for real-time PCR were as follows: forward, TAATCCCAGCGCTTTGGAAG; reverse, TTGCTAGATCCAGGTCTCTGCA for the upstream region of the Bax gene.

### Immunofluorescence

Cells were fixed in 3.7% formaldehyde in PBS for 10 min. After rinsing twice with PBS, the cells were permeabilized in 0.1% Triton X-100 in PBS and later blocked with TBS-T buffer containing 0.5% bovine serum albumin and 10% goat serum for 1 h at room temperature. Subsequently, the cells were incubated with anti-MYBBP1A and anti-p53 antibodies for 1 h, stained with Alexa Fluor-conjugated secondary antibodies (Invitrogen, Carlsbad, CA) for 1 h, and mounted with Vectashield (Vector Laboratories, Burlingame, CA). Immunofluorescent images were obtained by Biozero immunofluorescence microscopy (Keyence, Osaka, Japan).

## Results

### MYBBP1A expression decreases as breast cancer carcinogenesis progresses

**Figure 1:**
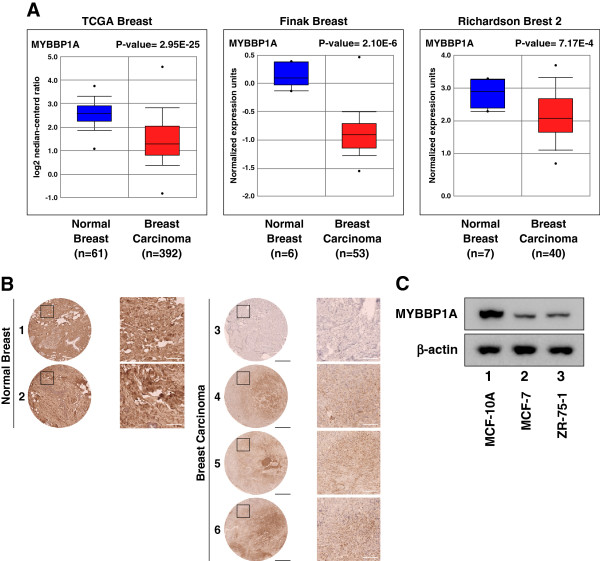
**MYBBP1A expression decreases as breast cancer carcinogenesis progresses.** (**A**) Decreased expression of *MYBBP1A* mRNA in breast carcinomas from three independent datasets for human breast cancers. Data and statistics were obtained from the Oncomine database [20-22,25]. (**B**) Decreased MYBBP1A protein expression in carcinoma samples of breast cancer patients. IHC was used to examine MYBBP1A expression in TMAs and compare it with that of human normal breast tissues and human breast cancer tissues. Counterstaining used hematoxylin. Representatives of normal breast tissue (1 and 2) and breast carcinoma tissue (3 to 6) are shown. Sample 3 is an infiltrating duct carcinoma of T2N1aM0, sample 4 is an infiltrating duct carcinoma of T3N2aM0, sample 5 is an infiltrating duct carcinoma of T2N0M0, and sample 6 is an infiltrating duct carcinoma of T2N0M0. The areas within the black squares are enlarged and shown in the right-side panels. Black scale bars = 0.5 mm and white scale bars = 100 μm. (**C**) MYBBP1A protein expression levels in MCF-10A, MCF-7, and ZR-75-1 cells. MYBBP1A protein levels in the cells were determined by immunoblottin.

We previously reported that MYBBBP1A was involved in activating p53 function. Therefore, we assumed that MYBBP1A would have a cancer prevention function via p53. To examine the relationship between *MYBBP1A* expression and breast cancer progression, we examined the *MYBBP1A* expression profiles in breast carcinomas compared to those of normal tissue using the Oncomine database, which provides publicly available datasets of gene expression in cancer. Of the 12 datasets, 11 contained gene chip profiles classified as normal or breast carcinoma tissues, which indicated that *MYBBP1A* mRNA levels were significantly lower in breast carcinomas than in normal tissues. Three representative results from independent datasets characterized by large population sizes are shown in Figure 1 (*MYBBP1A* expression levels in normal vs. breast carcinoma; P = 2.95E-25, 2.10E-6, and 7.17E-4). Next, we used IHC to assess MYBBP1A expression in non-neoplastic and breast cancer tissues using a human breast cancer tissue microarray. As shown in Figure 1B, MYBBP1A expression was significantly decreased in the breast cancer tumors. To confirm these results, we compared MYBBP1A protein levels in MCF-10A, MCF-7, and ZR-75-1 cells. The MYBBP1A protein levels were much lower in MCF-7 and ZR-75-1 cells, the cancer cell lines derived from human breast cancer cells, than in MCF-10A cells, a normal breast tissue cell line (Figure 1C). These results suggested that MYBBP1A had an inhibitory effect on carcinogenesis in these cancer patients.

### MYBBP1A suppresses colony formation and tumorigenesis *in vitro* and *in vivo*

**Figure 2:**
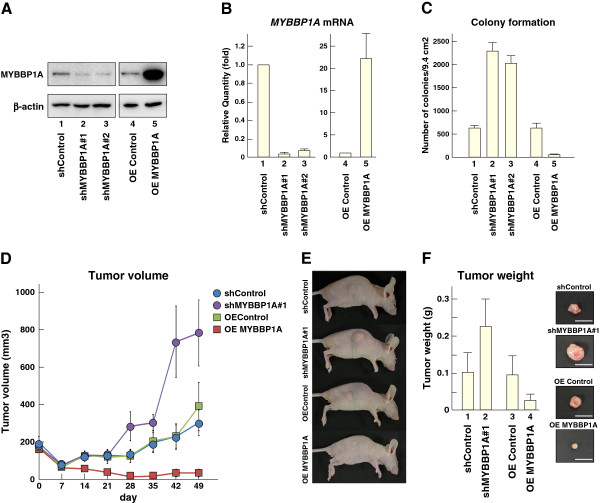
**MYBBP1A suppresses colony formation and tumorigenesis *in vitro *and *in vivo. ***(**A**) and (**B**) Efficiency of *MYBBP1A* knockdown and overexpression in MCF-7 cells. (**A**) western blot with anti-MYBBP1A antibody was used to detect MYBBP1A protein in MCF-7 cells that stably expressed shRNA for MYBBP1A (shMYBBP1A #1 and shMYBBP1A #2), cells that expressed Luciferase shRNA (shControl), cells that stably overexpressed MYBBP1A (OE MYBBP1A), and EGFP (OE Control). (**B**) *MYBBP1A* mRNA in these cells was quantified by RT–qPCR. (**C**) Effects of *MYBBP1A* expression on colony formation. Number of colonies per dish (area = 9.4 cm^2^) are shown. (**D**) Tumor growth curves in nude mice that were inoculated with either shControl, shMYBBP1A #1, OE Control, or OE MYBBP1A MCF‐7 cells. Nude mice received bilateral subcutaneous injections of control or cells. Tumor volume is presented as the mean ± s.d. (n = 10) for 5 mice in each group. (**E**) and (**F**) Increased or decreased tumor weight in mice injected with shMYBBP1A #1 or OE MYBBP1A cells. Photographs of mice (**E**) and tumors (**F**, right panels) are shown. Scale bars = 10 mm. Tumor weights after 49 days are shown in F, left panel. Bars = mean + s.d. (n = 10).

To investigate a possible relationship between MYBBP1A and breast cancer cell growth, we generated MCF-7 cells that had stably knocked down or overexpressed *MYBBP1A* (Figure 2A and 2B). MCF-7 is a breast cancer cell line that expresses wild type p53. As shown in Figure 2C, the colony numbers in soft agar were markedly increased when using *MYBBP1A* knockdown cells (shMYBBP1A #1 and shMYBBP1A sh#2). Conversely, *MYBBP1A* overexpression decreased the number of these colonies (Figure 2C: OE MYBBP1A).

Next, we performed xenograft experiments to test the effect of *MYBBP1A* expression on tumorigenicity *in vivo*. *MYBBP1A* knockdown cells formed tumors that were significantly larger than those of control cells. In contrast, *MYBBP1A* overexpressing cells formed smaller tumors than control cells (Figure 2D, 2E, and 2F). These results indicated that MYBBP1A could suppress breast cancer tumor growth.

### MYBBP1A induces anoikis in a p53-dependent manner

**Figure 3:**
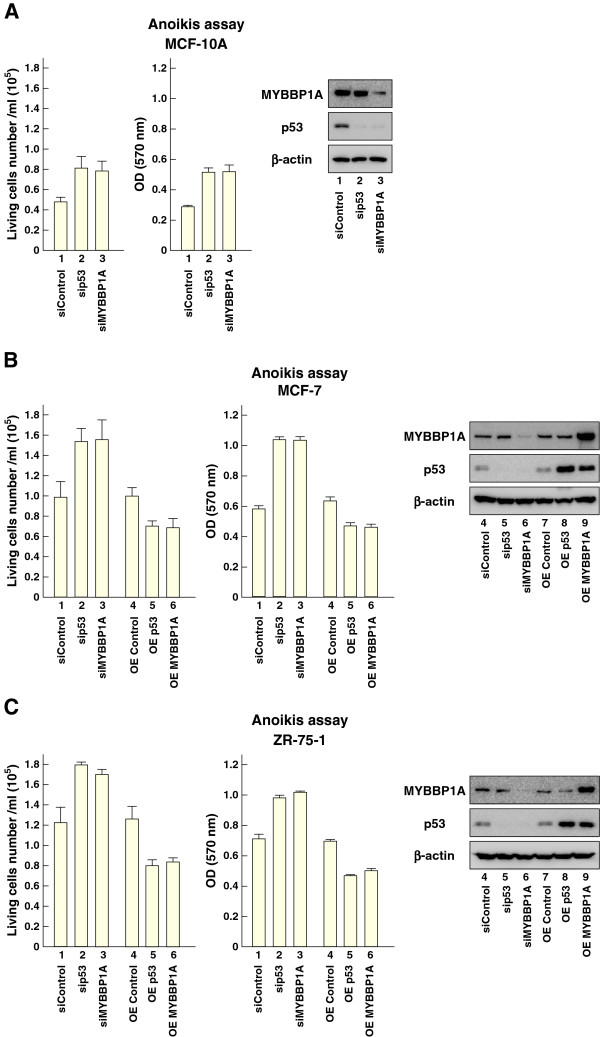
**MYBBP1A induces anoikis in normal breast and breast cancer cells. **(**A**) MYBBP1A induces anoikis in mammary epithelial cells. MCF-10A cells were transfected with siRNA for *p53* or *MYBBP1A*; 48 h later, anoikis assay was performed. These cells were cultured under suspension conditions for 24 h, and viable cell numbers were determined by trypan blue dye exclusion or MTT assay. Protein levels of MYBBP1A and p53 in the cells were determined by immunoblotting. (**B**) and (**C**) MCF-7 and ZR-75-1 cells were transfected with siRNA for 48 h, followed by anoikis assay, or expressed a plasmid for *p53* or *MYBBP1A* for 24 h followed by anoikis assay. These cells were cultured under suspension conditions for 24 h, and viable cell numbers were counted or assessed by MTT assay. Protein levels of MYBBP1A and p53 in these cells were determined by immunoblotting. Bars = mean + s.d. (n = 3).

To examine how MYBBP1A could suppress tumor formation we focused on anoikis, because p53 plays a critical role in anoikis [7-12]. Thus, we examined whether MYBBP1A was involved in anoikis in the context of the p53 pathway. MCF-10A mammary epithelial cells were transfected with sip53 or siMYBBP1A and cultured in non-adherent plates for 24 h. The number of viable cells under detached conditions increased with p53 or *MYBBP1A* knockdown (Figure 3A). MCF-7 and ZR-75-1 breast cancer cells were also cultured under detached conditions. The number of viable cells increased with *p53* knockdown, while they decreased when *p53* was overexpressed. Similarly, *MYBBP1A* knockdown increased and *MYBBP1A* overexpression decreased the numbers of viable cells under detached conditions (Figure 3B and 3C). These results indicated that MYBBP1A was involved in anoikis in breast tissues.

**Figure 4:**
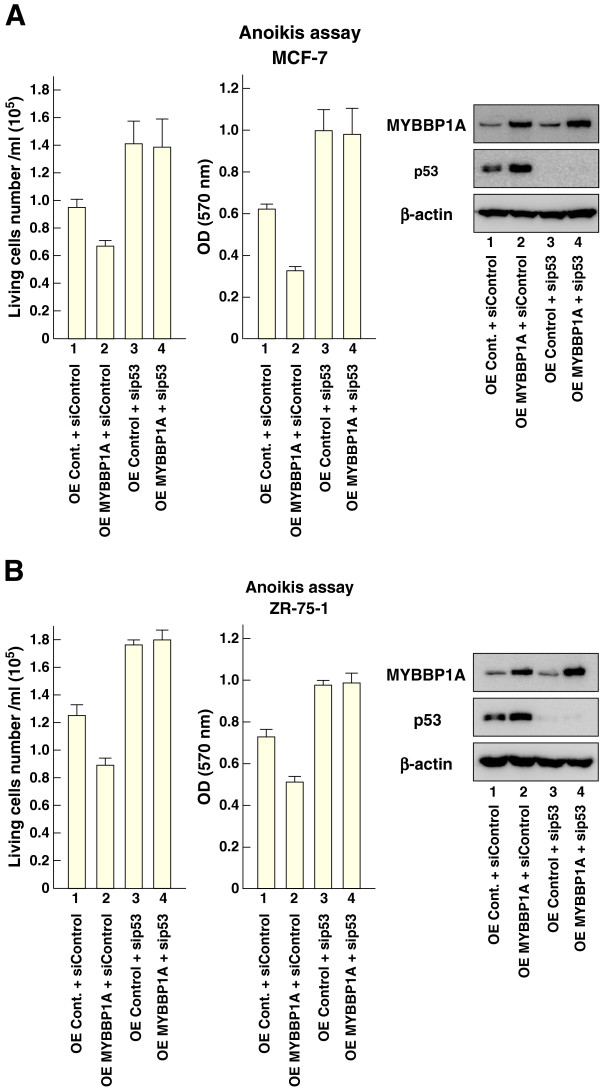
**MYBBP1A induces anoikis in a p53-dependent manner.** (**A**) and (**B**) p53 is required for MYBBP1A-induced anoikis. As indicated, MCF-7 and ZR-75-1 cells were transfected with a plasmid for *MYBBP1A* expression with or without siRNA for p53. These cells were cultured under suspension conditions for 24 h, and viable cell numbers were counted using trypan blue dye exclusion or assessed by MTT assay. Protein levels of MYBBP1A and p53 in the cells were determined by immunoblotting. Bars = mean + s.d. (n = 3).

To confirm that MYBBP1A was involved in anoikis in context of p53 activation, we tested the combination of *p53* knockdown and *MYBBP1A* overexpression in anoikis assay using MCF-7 cells (Figure 4A). Based on previous results (Figure 3B), *MYBBP1A* overexpression decreased the number of viable cells (compare lanes 1 and 2 in Figure 4A). However, *MYBBP1A* overexpression in *p53* knocked-down cells did not show any significant effects (compare lanes 3 and 4 in Figure 4A). Similar results were obtained in ZR-75-1 cells (Figure 4B).

**Figure 5:**
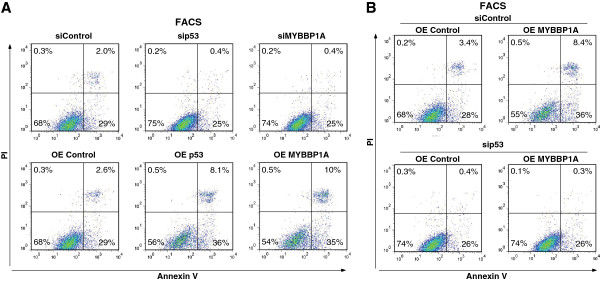
**MYBBP1A induces apoptosis during detached conditions in a p53-dependent manner.** (**A**) MCF-7 cells were transfected with siRNA or a plasmid for *p53* or *MYBBP1A* expression for 48 h, followed by anoikis assay. These cells were cultured under suspension conditions for 24 h, and apoptotic cells were determined by Annexin V–FITC/PI staining followed by flow cytometry analyses. (**B**) p53 is required for MYBBP1A-induced anoikis. As indicated, MCF-7 cells were transfected with a plasmid for *MYBBP1A* expression with or without siRNA for *p53*. They were then cultured under suspension conditions for 24 h, after which apoptotic cells were determined by Annexin V–FITC/PI staining and flow cytometry analyses.

To further examine the role of MYBBP1A in anoikis, we examined cellular apoptosis as determined by Annexin V–FITC/PI staining, followed by flow cytometric analysis. In accordance with the results shown in Figures 3 and 4, apoptosis decreased after *p53* and *MYBBP1A* knockdown and increased when these genes were overexpressed (Figure 5A). Moreover, *MYBBP1A* overexpression in *p53* knocked-down cells did not result in any significant effects (Figure 5B). These results indicate that MYBBP1A regulates p53-dependent anoikis.

### MYBBP1A enhances p53 target genes expression during anoikis

**Figure 6:**
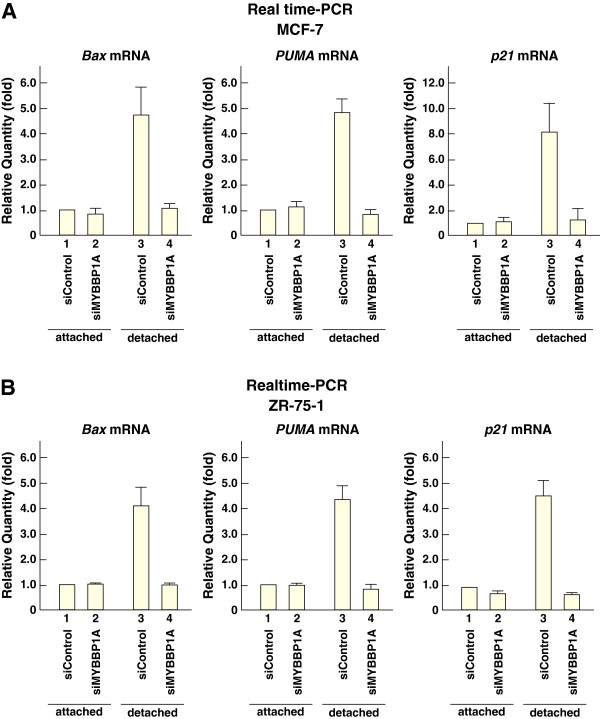
**MYBBP1A enhances p53 target genes’ expression during anoikis.** (**A**) and (**B**) MCF-7 and ZR-75-1 cells were treated with siRNAs for 48 h prior to anoikis assay and cultured under attached or detached conditions for 2 h (*Bax* mRNA) or 4 h (*PUMA* and *p21* mRNA). Total RNAs were prepared and expressions of the indicated genes were analyzed by RT–qPCR. Bars = mean + s.d. (n = 3).

Next, the effects of *MYBBP1A* knockdown on the induction of p53-target genes were examined. The mRNA levels of *Bax*, *PUMA*, and *p21* were increased under detached conditions, whereas the increases in these mRNA levels were suppressed when *MYBBP1A* was knocked down using siRNA in MCF-7 cells (Figure 6A). Similar results were obtained with ZR-75-1 cells (Figure 6B). These results suggest that MYBBP1A regulates p53-dependent anoikis by enhancing p53 activation.

### MYBBP1A enhances p53 activation during anoikis

**Figure 7:**
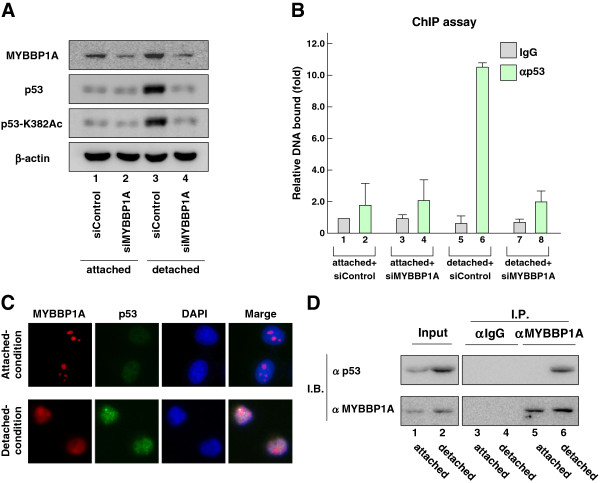
**MYBBP1A enhances p53 activation during anoikis.** (**A**) Acetylation at lysine 382 residues of p53 induced under detached conditions requires MYBBP1A. MCF-7 cells were treated with siControl or siMYBBP1A for 48 h followed by culture under attached or detached conditions for 2 h. Cell lysates were analyzed by immunoblot using the indicated antibodies. (**B**) p53 recruitment to the *Bax* promoter under detached conditions requires MYBBP1A. MCF-7 cells were treated as shown in (**A**), and ChIP assay was performed using normal mouse IgG and anti-p53 antibodies. The p53-binding region of the *Bax* promoter was amplified and analyzed by RT–qPCR. (**C**) MYBBP1A translocates from the nucleolus to the nucleoplasm under detached conditions. MCF-7 cells were cultured under attached or detached conditions for 2 h, after which the localizations of MYBBP1A and p53 were visualized by immunofluorescence using anti-MYBBP1A and anti-p53 antibodies. (**D**) Endogenous MYBBP1A associates with p53 under detached conditions. MCF-7 cells were cultured under detached conditions for 2 h. The cell lysates were immunoprecipitated with normal rabbit IgG or anti-MYBBP1A antibodies and analyzed by immunoblot using antibodies against MYBBP1A and p53. Bars = mean + s.d. (n = 3).

The acetylation levels of p53 are increased in response to stress and correlate well with p53 activation and stabilization [26-28]. Accumulating evidence supports the conclusion that acetylation stabilizes p53 and is indispensable for p53 activation [29,30]. Therefore, to study the molecular mechanism by which MYBBP1A induced anoikis in a p53-dependent manner, we examined whether MYBBP1A was involved in the accumulation of p53 protein and the acetylation of p53 K382 under detached conditions. Immunoblotting revealed that detached conditions induced the accumulation and acetylation of p53 (Figure 7A lane 3). However, p53 accumulation and acetylation were not observed when *MYBBP1A* was knocked down using siRNA (Figure 7A lane 4). This suggested that MYBBP1A was required for p53 activation in anoikis.

Consistent with these results, p53 recruitment to the *Bax* promoter was significantly enhanced under detached conditions, while p53 recruitment was abrogated by *MYBBP1A* knockdown (Figure 7B).

A previous report showed that MYBBP1A activates p53 by facilitating its direct interaction with p53 in response to stress when MYBBP1A translocated from the nucleolus to the nucleoplasm [13]. Therefore, we examined the localization of MYBBP1A and the interaction between MYBBP1A and p53 under detached conditions. Immunostaining revealed that MYBBP1A translocated from the nucleolus to the nucleoplasm under detached conditions (Figure 7C). Moreover, co-immunoprecipitation showed that endogenous MYBBP1A was bound to p53 in MCF-7 cells under detached conditions (Figure 7D). These results indicated that, under detached conditions, MYBBP1A translocates from the nucleolus to the nucleoplasm, and then binds to p53. Thus, MPBBP1A enhances p53 target gene transcription.

## Discussion

In this study, we revealed the physiological significance of MYBBP1A in p53 activation for prevention of cancer. MYBBP1A was originally identified as a protein that interacted with the negative regulatory domain of c-Myb [31]. However, in studies done by a number of different groups, MYBBP1A was found to interact with and regulate several transcription factors. MYBBP1A binds to Prep1 or PGC-1α and inhibits their activity. Prep1 is involved in development and organogenesis, and PGC-1α is a key regulator of metabolic processes such as mitochondrial biogenesis, respiration, and gluco neogenesis in the liver [32,33]. Correspondingly, MYBBBP1A also interacts with NF–κB and CRY1 and regulates their transcriptional activity [15,34].

We previously reported that MYBBP1A interacts with p53 and activates its transcriptional capacity. MYBBP1A localizes predominantly in the nucleolus; however, it translocates from the nucleolus to the nucleoplasm in response to cellular stress and activates p53 [13,14,35]. Similar to other kinds of stress, detached conditions induce p53 acetylation and target gene transcription in an MYBBP1A-dependent manner (Figures 6 and 7A). MYBBP1A plays an important role in p53 activation in response to detached condition to induce anoikis.

The regulation of MYBBP1A localization under detached conditions is still to be elucidated. When cells are exposed to UV light, MYBBP1A translocation is accompanied by nucleolar segregation [13]. Because the nucleolus appears to be intact after 2 h under detached conditions, there may be another signal that releases MYBBP1A from the nucleolus.

Numerous reports have shown that other nucleolar proteins can activate p53 similar to that by MYBBP1A. RPL11 directly bind to HDM2 and inhibit HDM2-mediated p53 ubiquitination [36-41]. Similarly, the nucleolar proteins NPM, NCL, NS, and ARF can also directly bind to HDM2 and inhibit p53 ubiquitination [42-46]. Unlike with these nucleolar factors, MYBBP1A promotes p53 activation by directly binding to p53 without affecting HDM2 function. With regard to the pronounced effect of MYBBP1A knockdown on p53 accumulation and acetylation under detached conditions (Figure 7A), MYBBP1A may have a unique and essential function in response to detached conditions.

In addition, Sanhueza *et al.* recently reported that MYBBP1A regulates the proliferation and migration of head and neck squamous cell carcinoma cells [17]. However, the detailed mechanisms underlying these activities are unknown. In this study, we revealed a function for the nucleolar protein MYBBP1A in breast cancer. Therefore, our results provide a novel insight into the function of the nucleolar protein MYBBP1A in the biology of cancer cells.

## Conclusion

**Figure 8:**
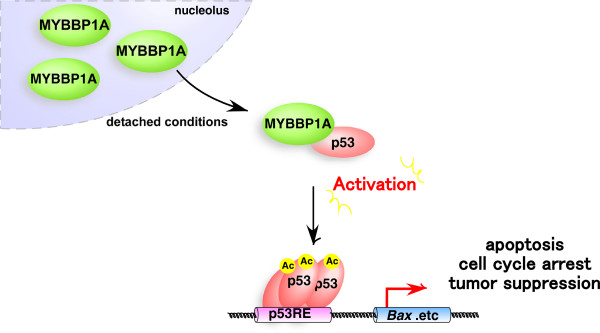
**Proposed model for the role of MYBBP1A in p53 dependent anoikis.** See the text.

To determine the role of MYBBP1A in cancer, we conducted an extensive analysis of the Oncomine database and IHC studies, and showed that *MYBBP1A* expression was associated with breast cancer tumorigenesis. *In vivo* and *in vitro* experiments using the breast cancer cell lines revealed that tumorigenesis, colony formation, and anoikis resistance were significantly enhanced by *MYBBP1A* knockdown*.* Co-immunoprecipitation experiments revealed that MYBBP1A binds to p53 under detached conditions and enhances p53 target gene transcription (Figure 8). These results revealed the physiological significance of MYBBP1A in p53 activation. Our results may lead to a novel strategy for breast cancer therapy.

## Abbreviations

p53: Protein 53; MYBBP1A: Myb-binding protein 1a; ECM: Extracellular matrix; p21: Protein 21; Bax: Bcl-2–associated X protein; PUMA: p53 upregulated modulator of apoptosis; p300: Protein 300; IHC: Immunohistochemistry; FACS: Fluorescence-activated cell sorting; FITC: Fluorescein isothiocyanate; RT-qPCR: Reverse transcription quantitative polymerase chain reaction; DMEM: Dulbecco’s modified Eagle’s medium; FBS: Fetal bovine serum; PCR: Polymerase chain reaction; shRNA: Short hairpin RNA; siRNA: Small interfering RNA; RT-PCR: Reverse transcription polymerase chain reaction; PMSF: Phenylmethylsulfonyl fluoride; SDS-PAGE: Sodium dodecyl sulfate-polyaclylamidegel electrophoresis; ECL: Enhanced chemiluminescence; ChIP: Chromatin immunoprecipitation; PBS: Phosphate buffered saline; TBS-T: Tris-buffered saline and tween 20; TMA: Tissue microarray; OE: Overexpression; CBP: CREB-binding protein; qPCR: Quantitativepolymerase chain reaction; PGC-1α: Peroxisome proliferator-activated receptor gamma coactivator 1-alpha; NF-κB: nuclear factor kappa-light-chain-enhancer of activated B cells; CRY1: Cryptochrome 1.

## Competing interests

The authors declare that they have no conflicts of interests concerning this work.

## Authors’ contributions

KA participated at the design, execution and interpretation of the experiments, as well as writing up of the manuscript. WO participated at the immunoblotting experiments and the presentation of the manuscript. YH participated at the FACS analysis. HK participated at the interpretation of the data and the presentation of the manuscript. JY participated at the design and interpretation of the experiments, as well as writing up of the manuscript. All authors read and approved the final manuscript.

## Pre-publication history

The pre-publication history for this paper can be accessed here:

http://www.biomedcentral.com/1471-2407/13/65/prepub
